# Evaluation of the Aquatic Toxicity of Several Triazole Fungicides

**DOI:** 10.3390/metabo14040197

**Published:** 2024-04-02

**Authors:** Bianca-Vanesa Boros, Diana-Larisa Roman, Adriana Isvoran

**Affiliations:** 1 Department of Biology-Chemistry, Faculty of Chemistry, Biology, Geography, West University of Timisoara, 16 Pestalozzi, 300115 Timisoara, Romania; bianca.boros@e-uvt.ro (B.-V.B.); diana.roman@e-uvt.ro (D.-L.R.); 2 Advanced Environmental Research Laboratories (AERL), 4 Oituz, 300086 Timisoara, Romania

**Keywords:** *Lemna minor*, aquatic organisms, photosynthesis

## Abstract

Fungicides play an important role in crop protection, but they have also been shown to adversely affect non-target organisms, including those living in the aquatic environment. The aim of the present study is to combine experimental and computational approaches to evaluate the effects of flutriafol, metconazole, myclobutanil, tebuconazole, tetraconazole and triticonazole on aquatic model organisms and to obtain information on the effects of these fungicides on *Lemna minor*, a freshwater plant, at the molecular level. The EC_50_ (the half-maximum effective concentration) values for the growth inhibition of *Lemna minor* in the presence of the investigated fungicides show that metconazole (EC_50_ = 0.132 mg/L) and tetraconazole (EC_50_ = 0.539 mg/L) are highly toxic, tebuconazole (EC_50_ = 1.552 mg/L), flutriafol (EC_50_ = 3.428 mg/L) and myclobutanil (EC_50_ = 9.134 mg/L) are moderately toxic, and triticonazole (EC_50_ = 11.631 mg/L) is slightly toxic to this plant. The results obtained with the computational tools TEST, ADMETLab2.0 and admetSAR2.0 also show that metconazole and tetraconazole are toxic to other aquatic organisms: *Pimephales promelas*, *Daphnia magna* and *Tetrahymena pyriformis*. A molecular docking study shows that triazole fungicides can affect photosynthesis in *Lemna minor* because they strongly bind to C43 (binding energies between −7.44 kcal/mol and −7.99 kcal/mol) and C47 proteins (binding energies between −7.44 kcal/mol and −8.28 kcal/mol) in the reaction center of photosystem II, inhibiting the binding of chlorophyll a to these enzymes. In addition, they can also inhibit glutathione S-transferase, an enzyme involved in the cellular detoxification of *Lemna minor*.

## 1. Introduction

Pesticides are chemical substances widely used in agriculture to control pests, weeds and plant diseases, and their global consumption was about 4.3 million metric tons in 2023; this number is expected to grow in the next few years to a value of approximately 4.41 million metric tons in 2027 [1]. Excessive use of pesticides is already known to negatively affect aquatic ecosystems, usually due to the runoff of pesticides used in agriculture [2]. Among pesticides, fungicides are important pollutants of the aquatic environment because they can enter these ecosystems through several pathways and produce toxic effects to a broad range of aquatic organisms [3]. In 2022, the global market of fungicides was evaluated at USD 20.8 billion and it is expected to increase to USD 28 billion by 2027 [4].

One of the most frequently used fungicide classes worldwide are azoles (imidazoles and triazoles), as they are the active ingredients in numerous pharmaceutical products and pesticides. They are recognized as environmental contaminants, and it has already been shown that they affect the quality of groundwater, surface waters and even drinking water [5]. The molecular properties of triazole fungicides used in agricultural practices reveal that they have low molecular weight and are moderately lipophilic, making them easily absorbable and persistent in sediments and organic surfaces in aquatic ecosystems [3,6]. The high persistence indicates that they may be found for long period of time in sediments, and, in that way, there is an increase of exposure duration locally at low concentrations and also potentially downstream due to sediment remobilization. Consequently, triazole fungicides are known to negatively affect numerous non-target organisms found in water and soil (and, accordingly, the activities of soil enzymes) [6,7,8,9,10] and even human health [10,11,12,13].

The data from the literature reveal that fungicides can affect plant physiology, particularly by influencing plant metabolic processes, including photosynthesis (by inhibiting photosystems and causing gradual chlorosis), inhibition of antioxidant-defense enzyme activities, pigment biosynthesis and root growth [14,15,16]. As for triazole fungicides, depending on crops and doses, they can stimulate or reduce photosynthesis [14,16,17,18]. Yang and coworkers [19] shown that prothioconazole, a triazole fungicide, revealed toxicity against *Lemna minor* by inhibiting the biosynthesis of photosynthetic pigments and the activity of antioxidant-defense enzymes. Another study revealed that clotrimazole, an imidazole fungicide, also caused toxicity in *L. minor* by reducing the pigment contents, and high concentrations of the fungicide also caused an increase in catalase activity, and a decrease in glutathione S-transferase activity [20].

The hypothesis of this study is that triazole fungicides may affect various organisms living in aqueous environments. Consequently, it focuses on evaluating the effects of several triazole fungicides (flutriafol, metconazole, myclobutanil, tebuconazole, tetraconazole and triticonazole) on model aquatic organisms. Experimental testing of the effects of fungicides on all individual aquatic species is difficult to achieve. From this point of view, computational studies are promising tools to predict missing data. Quantitative structure–toxicity studies of chemicals have been shown to be able to predict fungicides’ toxicity in aquatic indicator species [21,22]. Despite these encouraging results, studies modeling the effects of fungicides on aquatic ecosystems are scarce. Therefore, the aim of this study is to combine experimental and computational methods to evaluate the effects produced by the investigated triazole fungicides on several aquatic model organisms. The experimental approach aims to evaluate the effects of triazole fungicides on *Lemna minor*, a freshwater aquatic plant species. The computational approach is based on the prediction of the toxicity of the investigated fungicides on several model organisms in the aqueous environment. In addition, a molecular docking study was considered to assess the possible effects of fungicides at the molecular level by evaluating their interactions with enzymes involved in photosynthetic systems, in redox control and cellular detoxification.

As aquatic resources are precious natural assets, our study is significant as it assesses the effects of contamination of the aquatic environment with triazole fungicides and provides information that should be used to adopt appropriate crop management and measures leading to the responsible application of triazole fungicides. Furthermore, this study contributes to the understanding of the molecular mechanisms triggered by triazole fungicides and provides a comprehensive understanding of the pathways through which they manifest their toxicity on *Lemna minor*.

## 2. Materials and Methods

### 2.1. Materials Used in the Experimental Approach

**Figure 1:**
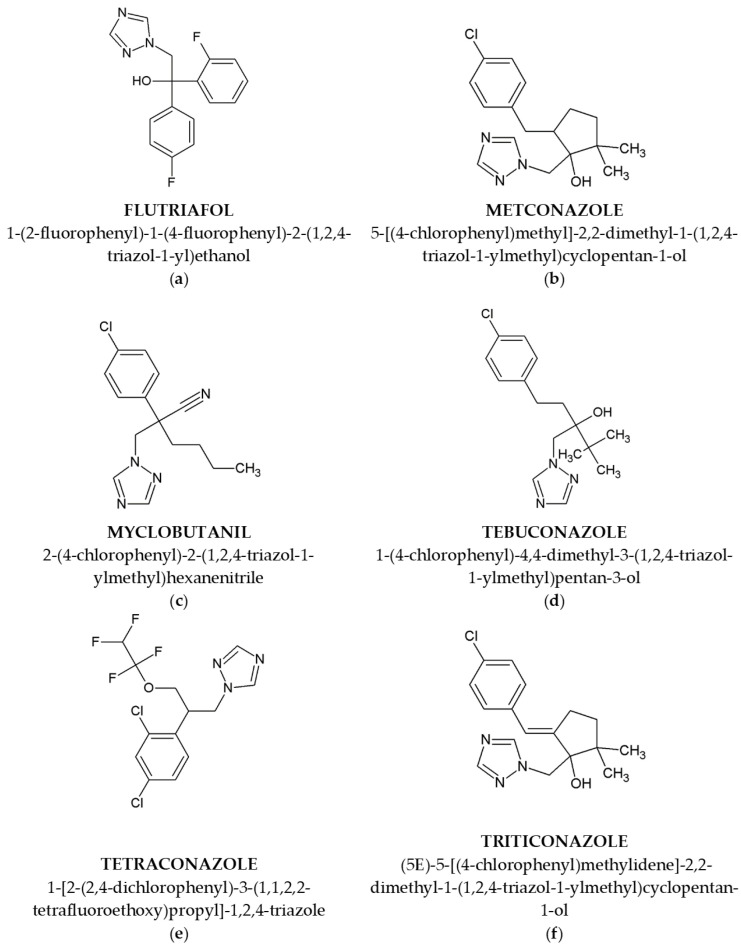
Triazole fungicides considered in this study—2D formulas, common and IUPAC names: (**a**) flutriafol; (**b**) metconazole; (**c**) myclobutanil; (**d**) tebuconazole; (**e**) tetraconazole; (**f**) triticonazole.

Six types of fungicides were tested, namely flutriafol, metconazole, myclobutanil, tebuconazole, tetraconazole and triticonazole (Figure 1).

After prior range-finding tests (data not shown), five concentrations were selected for each fungicide. Test solutions were prepared by diluting the commercial product (Table 1) in culture media. When computing EC_50_ values, the concentration of active substance in these products was taken into account, and these concentrations are presented in Table 1.

Zinc chloride 0.5% (Order No. 3533) was purchased from Carl Roth (Karlsruhe, Germany) and used as the positive control. Duckweed culture media was prepared according to the OECD guideline for a *Lemna minor* ecotoxicity assay [23], and all chemicals used were reagent grade.

### 2.2. Lemna minor Growth Inhibition Assay

The common duckweed, *Lemna minor*, was used as a test organism to analyze the growth response of the tested fungicides using a growth inhibition study. Both the *L. minor* culture and the growth inhibition assay were carried out under standard conditions as indicated in the OECD guidelines [23].

Ten fronds per test vessel were used to investigate the effects of the triazole fungicides over the course of a seven-day exposure period. Two controls were examined under identical circumstances: 0.5% zinc chloride served as the positive control, while culture media served as the negative control. All experiments were done in triplicate and measurements were performed by the same researcher on the same day. Similar duckweed growth inhibition tests have been performed in the past for chitosan [24] and alginate [25].

The number of fronds served as the endpoint for the growth inhibition test, and this number was used to plot dose–response curves and determine the half-maximum effective concentration (EC_50_). Based on the computed EC_50_ values, the tested samples were categorized into the following aquatic ecotoxicity categories by the U.S. Environmental Protection Agency: very highly toxic (<0.1 mg/L), highly toxic (0.1–1 mg/L), moderately toxic (>1–10 mg/L), slightly toxic (>10–100 mg/L) and practically non-toxic (>100 mg/L) [26]. Computed EC_50_ values, and consequently the aquatic ecotoxicity categories, have been compared for every fungicide with other ecotoxicity published data and/or with data deposited in Pesticides Properties Data Base (PPDB) [27].

### 2.3. Statistical Analysis Used in the Experimental Approach

For the statistical analysis of the data, PAST software, version 4.16 [28] was utilized, and the dose–response curves, confidence intervals and EC_50_ values were obtained with OriginPro software (OriginPro Version 2021, OriginLab Corporation, Northampton, MA, USA). For testing the data’s normality, the Shapiro–Wilk W test was applied. After analyzing the distribution, an ANOVA analysis was performed. Parametric tests were utilized to assess the normally distributed data, Levene’s test was used to determine whether the variation among treatments was homogeneous, and Tukey’s post hoc test was used to analyze variances. The Kruskal–Wallis test was used to assess the non-normally distributed data; Dunn’s post hoc analysis was then used to examine variances. The differences were considered significant for *p* < 0.05.

### 2.4. Predictions of the Toxicological Effects of Triazole Fungicides on Aqueous Organisms

TEST 5.1.1 (Toxicity Estimation Software Tool) [29], ADMETLab2.0 [30] and admetSAR2.0 [31,32] computational tools were utilized in this study to obtain predictions regarding the toxicity of investigated fungicides against aquatic organisms. For all these computational tools, the query molecule was represented using Simplified Molecular Input Line Entry System (SMILES) notation. The canonical SMILES notations for the investigated fungicides were retrieved from the PubChem database [33].

TEST 5.1.1 is a computational tool developed by the United States Environmental Protection Agency (U.S. EPA). It is used to predict the toxicological properties of investigated fungicides against organisms living in an aqueous environment starting from their molecular structures and using the QSAR (Quantitative Structure Activity Relationship) methodology. The following toxicological endpoints are predicted using TEST software: (i) 96 h LC_50_ (lethal concentration 50, the amount of a substance required to kill 50% of a test organism) for fathead minnow (*Pimephales promelas*) expressed as −log_10_ (mol/L) (goodness-of-fit is R^2^ = 0.729); (ii) 48 h LC_50_ for *Daphnia magna* expressed as −log_10_ (mol/L) (R^2^ = 0.616); and (iii) IGC_50_ (50% growth inhibitory concentration) for *Tetrahymena pyriformis* expressed as −log_10_ (mol/L) (R^2^ = 0.739) [29].

The ADMETLab2.0 online server is able to create predictions regarding the ADMET (Absorption Distribution Metabolism Excretion Toxicity) profiles of various chemicals and includes predictions of ecotoxicity data like 48 h IGC_50_TP (concentration of the investigated chemical in water that causes 50% growth inhibition to *Tetrahymena pyriformis* after 48 h) expressed as −log_10_[(mg/L)/(1000 × MW)] (R^2^ = 0.860), 96 h fathead minnow (*P. promelas*) LC_50_ (LC_50_FM) expressed as −log_10_[(mg/L)/(1000 × MW)] (R^2^ = 0.660), and 48 h *Daphnia magna* LC_50_ (LC_50_DM) expressed as −log_10_[(mg/L)/(1000 × MW)] (R^2^ = 0.909) [30].

The admetSAR2.0 online server (http://lmmd.ecust.edu.cn/admetsar2/ accessed on 17 January 2024) provides predictions of ADMET and eco-toxicological profiles for various chemical compounds [31,32]. In the present study, the probabilities that the investigated fungicides produce toxicity against fish, fathead minnow (*P. promelas*) and crustaceans (*Daphnia magna*), as well as the IGC_50_ values for *Tetrahymena pyriformis*, are calculated. The prediction of toxicity against fathead minnow is based on a qualitative classification model containing 554 molecules (366 reveal toxicity to this organism and 118 are non-toxic) and has an accuracy of 83.9%. Similarly, the prediction of toxicity to crustaceans is based on another qualitative classification model containing 660 molecules (336 reveal toxicity to this organism and 324 are non-toxic) and has an accuracy of 76.6%. The prediction regarding the toxicity towards *Tetrahymena pyriformis* is based on a regression model containing 1571 molecules and which allows the estimation of the IGC_50_ values for these organisms due to the presence of the chemical substance (R^2^ = 0.822) [32].

### 2.5. Molecular Docking Study

Pesticides are known to affect photosynthesis by binding to specific sites within the photosystem II complex in plant chloroplasts and cause gradual chlorosis in plants, followed by necrosis of leaf tissue [34]. Furthermore, it was revealed that the triazole fungicide prothioconazole inhibited the biosynthesis of photosynthetic pigments and the activity of antioxidant defense enzymes in *Lemna minor* [19] and that clotrimazole, an imidazole fungicide, caused a decrease in glutathione S-transferase activity [20]. Starting from this information, in this study, the molecular docking method was considered to evaluate the interactions of the investigated triazole fungicides with the main enzymes involved in photosynthesis systems, redox control and cellular detoxification processes in *Lemna minor.* Consequently, the molecular docking of every investigated fungicide with the following enzymes was considered: (i) chloroplast ATP synthase subunit alpha and chloroplast ATP synthase subunit beta as enzymes involved in ATP production during photosynthesis [35]; (ii) photosystem I P700 chlorophyll a apoproteins A1 and A2 as the major subunits of photosystem I and binding the majority of chlorophyll a, phylloquinone molecules and carotenoids [36,37,38]; (iii) photosystem II CP43 and CP47 chlorophyll a and beta-carotene binding proteins [39,40]; (iv) photosystem II reaction center proteins D1 and D2 [39,40]; (v) the ribulose bisphosphate carboxylase large chain that is responsible for starting the Calvin cycle by fixation of atmospheric CO_2_ to ribulose-1,5-bisphosphate in order to obtain two molecules of 3-phosphoglycerate [41]; (vi) glutathione peroxidase that catalyzes the conversion of organic hydroperoxides that are involved in redox control [42]; and (vii) glutathione S-transferase that catalysis glutathione-dependent processes involving xenobiotics resulting in cellular detoxification [43]. As there are not solved three dimensional structures for these enzymes belonging to *Lemna minor*, the AlphaFold structural models [44] were considered for docking. To identify the catalytic sites of these enzymes and to analyze the docking outputs, structural files for similar enzymes belonging to other plants and having solved three dimensional structures were considered. The AlphaFold models and the corresponding structural files, extracted from Protein Data Bank (PDB) [45] and considered in this study, are shown in Table 2 along with the root mean square deviation (RMSD) values obtained for superimposing the AlphaFold models and the crystallographic structures, and revealing the structural similarity between the models and the corresponding structures.

The superposition of the AlphaFold models for photosystem I P700 chlorophyll a apoproteins A1 and A2 revealed a high structural similarity of the two proteins (RMSD = 0.681 Å for all 574 pruned carbon alpha atom pairs, Supplementary Materials, Figure S1a) and, consequently, only one structure was considered for docking. A similar situation was registered for photosystem II reaction center proteins D1 and D2 (RMSD = 0.998 Å for all 248 pruned carbon alpha atom pairs, Supplementary Materials, Figure S1b). The structures of triazole fungicides were taken from PubChem [33].

Chimera software [46] was used to superimpose the structures, prepare the structures for docking and analyze the docking results. Molecular docking was implemented using the SwissDock server [47]. An accurate, rigid and blind docking was selected.

## 3. Results and Discussions

### 3.1. Effects of Triazole Fungicides on Lemna minor

**Figure 2:**
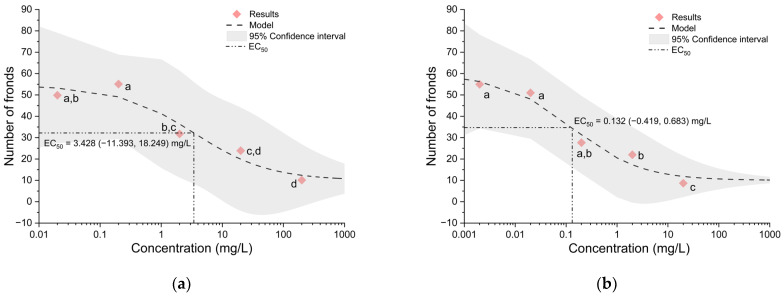
Dose–response curves and EC_50_ values of (**a**) flutriafol and (**b**) metconazole. The confidence intervals are shown as a grey area. Symbols that are marked with the same letters do not show statistically significant differences.

**Figure 3:**
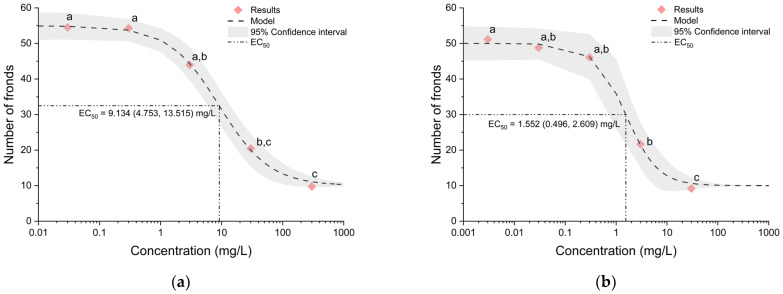
Dose–response curves and EC_50_ values of (**a**) myclobutanil and (**b**) tebuconazole. The confidence intervals are shown as a grey area. Symbols that are marked with the same letters do not show statistically significant differences.

**Figure 4:**
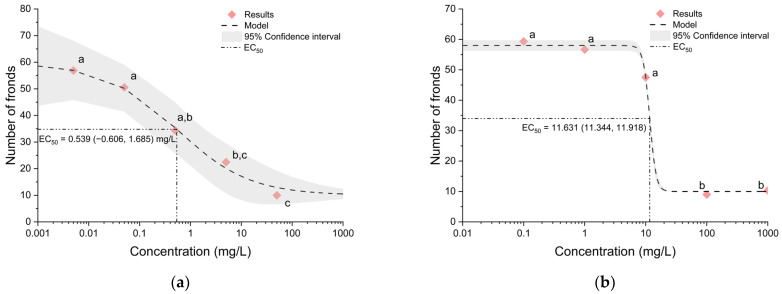
Dose–response curves and EC_50_ values of (**a**) tetraconazole and (**b**) triticonazole. The confidence intervals are shown as a grey area. Symbols that are marked with the same letters do not show statistically significant differences.

The results obtained by the exposure of common duckweed (*Lemna minor* L.) to the six tested fungicides allowed the plotting of dose–response curves, based on the concentration of fungicides and the total number of fronds, and the calculation of the half-maximal effective concentration (EC_50_) of each fungicide (Figure 2, Figure 3 and Figure 4).

The dose–response curve of flutriafol allowed the calculation of an EC_50_ value of 3.43 mg/L (Figure 2a), thus placing this fungicide in the moderately toxic category according to the EPA Ecotoxicity Categories for Aquatic Organisms [26]. In the case of metconazole, the calculated EC_50_ value was 0.13 mg/L (Figure 2b); thus, it falls into the highly toxic category.

There is a lack of information regarding the effects of flutriafol on *Lemna minor*, although there is information in the PPDB on *Lemna gibba*; the EC_50_ value is 0.65 mg/L [48], indicating a moderate toxicity against this aquatic plant. According to the PPDB, EC_50_ values for flutriafol have also been determined for other aquatic organisms (*Chironomus riparius*, *Daphnia magna*, *Pimephales promelas* and *Lepomis macrochirus*) and indicate moderate ecotoxicity (0.01–10 mg/L), excepting the EC_50_ value for the micro algae *Raphidocelis subcapitata* which reveals low toxicity [48].

Information is also missing regarding the effects of metconazole on *Lemna minor*. There is, however, information on the EC_50_ value on *Lemna gibba* in the PPDB. This value is 0.53 mg/L and classified metconazole as moderately toxic according to the PPDB. EC_50_ values for other aquatic organisms, such as *Raphidocelis subcapitata*, *Chironomus riparius*, *Daphnia magna* and *Oncorhynchus mykiss* are also available in the PPDB, all indicating the moderate aquatic toxicity of this fungicide [49].

According to this study, myclobutanil is classified as moderately toxic, with an EC_50_ value of 9.13 mg/L (Figure 3a), alongside tebuconazole, which is classified as moderately toxic with a calculated EC_50_ value of 1.55 mg/L (Figure 3b).

The effects of myclobutanil on *Lemna minor* have been addressed in the literature and the EC_50_ value determined was 1.89 mg/L [50], a value 5.4 times lower than that determined in this study. A possible difference in the two values of EC_50_ may be due to the fact that the above cited test was performed in 6-well microplates, using a number of four fronds per well, whereas in the current study, culture dishes with a depth greater than that of a microplate were used, with 10 fronds per vessel. Also, the duckweed was grown in Steinberg medium in the cited study [50], while in the current study Swedish Standard medium was used, according to OECD guidelines. EC_50_ values for myclobutanil were also determined for the alga *Scenedesmus obliquus*, with a value of 3.95 mg/L [51] showing moderate aquatic acute toxicity, and for the protozoan *Tetrahymena thermophila*, with a value of 14.31 mg/L showing a slight toxicity [52]. According to the PPDB, the EC_50_ value for *Lemna gibba* is greater than 105 mg/L, interpreted as representing low toxicity. Also in the PPDB, EC_50_ values that are specified for other aquatic organisms (*Scenedesmus subspicatus*, *Chironomus riparius*, *Americamysis bahia*, *Daphnia magna* and *Oncorhynchus mykiss*) indicate moderate toxicity of myclobutanil [53].

We could not identify any studies addressing the ecotoxicological effects of tebuconazole on *Lemna minor*. Studies on *Lemna gibba* have been conducted by both the US Environmental Protection Agency [54] and the European Food Safety Authority [55], with 14-day EC_50_ values for tebuconazole of 0.151 mg/L and 0.144 mg/L, respectively and classifying tebuconazole as moderately toxic. The effect of tebuconazole was also tested on another duckweed species, *Spirodela polyrhiza*, with a 72 h EC_50_ value of 2.204 mg/L. This value is close to that identified on *Lemna minor* in the present study, being only 1.3 times higher [56]. Ecotoxicity data on *L. gibba* are also given in the PPDB, with an EC_50_ value of 0.14 mg/L, corresponding to moderate toxicity. In this database, there is also ecotoxicological characterization for the aquatic organisms *Scenedesmus subspicatus*, *Chironomus riparius* and *C. dilutus*, *Mysidopsis bahia*, *Daphnia magna* and *Oncorhynchus mykiss*, showing moderate toxicity of this fungicide [57].

According to the dose–response curve of tetraconazole, the calculated EC_50_ value is 0.54 mg/L (Figure 4a), which classifies this fungicide as highly toxic. For the fungicide triticonazole, an EC_50_ value of 11.63 mg/L was calculated (Figure 4b), thus classifying it as slightly toxic.

To the best of our knowledge, there are no studies that have addressed the ecotoxicity of tetraconazole on *Lemna minor*, but there is information on another duckweed species, namely *L. gibba*, in the PPDB. The corresponding EC_50_ value is 0.52 mg/L, showing high toxicity. Also, in this database, there is information on the ecotoxicity of this fungicide on other aquatic organisms (*Ankistodesmus bibaiamus*, *Chironomus riparius*, *Mysidopsis bahia*, *Daphnia magna*, *Pimephales promelas* and *Lepomis macrochirus*), showing moderate toxic effects of tetraconazole on these organisms [58]. The ecotoxicological effects of tetraconazole were also determined on the algae *Pseudokirchneriella subcapitata*, with an IC_50_ value between 11 and 23 μg/L, and on the crustacean *Daphnia magna*, with an LC_50_ value between 5.2 and 10 μg/L; for both organisms, tetraconazole is considered as very highly toxic [59].

The effects of the triazole fungicide triticonazole have been previously studied on *Lemna minor*, and an EC_50_ value of 3.73 mg/L was determined [60]. This value is 3.5 times lower than that determined experimentally in this study. This difference may be due to the different testing conditions in the cited study and the current one. In the cited study, Petri dishes were used for testing, while in the current study, test dishes with greater depth were used. Also, in the quoted study, the test solution was renewed daily, while in the current study, the test solution was maintained throughout the 7-day test period. The literature data reveal the effect of triticonazole on other aquatic organisms, such as *Chlorella pyrenoidosa* (EC_50_ of 1.94 mg/L) [61], *Daphnia magna* (EC_50_ of 1.22 mg/L), *Danio rerio* (EC_50_ of 5.06 mg/L) and *Xenopus laevis* (EC_50_ of 8.06 mg/L) [60]; this fungicide is moderately toxic to these organisms. In the PPDB, there is data on the ecotoxicity of this fungicide on another duckweed species, *Lemna gibba*. The EC_50_ value is 1.1, which is lower than in this study. Data on *Pseudokirchneriella subcapitata*, *Chironomus riparius*, *Daphnia magna* and *Oncorhynchus mykiss* from the PPDB show moderate toxicity of triticonazole in these aquatic organisms [62].

**Figure 5:**
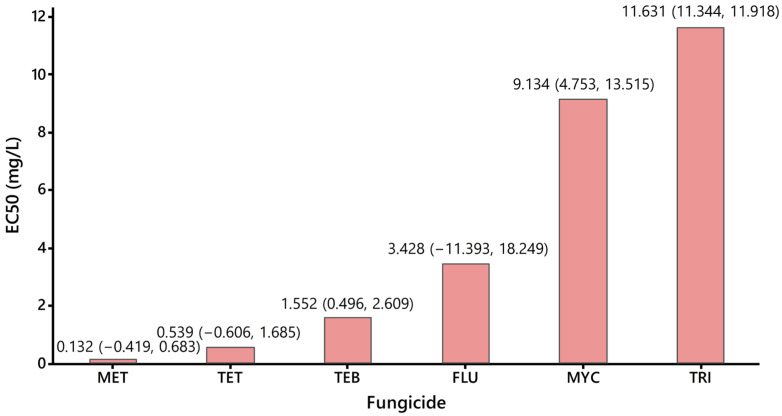
Comparison of EC_50_ values of tested fungicides: MET—metaconazole, TET—tetraconazole, TEB—tebuconazole, FLU—flutriafol, MYC—myclobutanil, TRI—triticonazole. The 95% confidence intervals are given in brackets (lower confidence limit, upper confidence limit).

The comparison of the EC_50_ values calculated for the six fungicides tested (Figure 5) revealed that the most toxic fungicide for *Lemna minor* is metconazole, followed by tetraconazole. The least toxic fungicide was found to be triticonazole, followed by myclobutanil. These affirmations are in very good agreement with data presented in the PPDB and the specific literature revealing that metconazole and tetraconazole emphasize high toxicity against numerous other aquatic organisms, whereas triticonazole and myclobutanil are only slightly toxic for other aquatic organisms.

As far as we know, this is the first study revealing the EC_50_ values for inhibition of *Lemna minor* for flutriafol, metconazole, tebuconazole and tetraconazole. As for the effect of other triazole fungicides on *Lemna minor*, these are diverse. Although several triazole fungicides have been tested on the common duckweed, EC_50_ values were calculated for only some of these, such as fluconazole (1.85 mg/L), propiconazole (1.07 mg/L) and prothioconazole (2.36 mg/L) [19,63]; these fungicides are moderately toxic according to the aquatic acute toxicity categories [26].

All the data resulting from the experiments carried out in this study are mostly in good qualitative agreement with other data presented in the specialized literature, which also emphasize the at least moderate toxicity of the fungicides tested against different types of aquatic organisms. There were quantitative differences that were recorded between the EC_50_ values for *Lemna minor* determined in this study in the presence of the investigated fungicides and the EC_50_ values for *Lemna minor* and/or for *Lemna gibba* (a member of the same subfamily) resulting from other studies in the presence of the same fungicides. These differences may be due to the distinct experimental approaches used, such as different exposure times, ambient temperatures, culture media, and experimental designs. This discrepancy emphasizes the need to apply standard test methods when evaluating ecotoxicity, which would allow comparison of results performed by different research groups, as standardized tests should provide similar results within the statistical limits of the method.

### 3.2. Prediction of Toxicity of Triazole Fungicides on Other Aqueous Organisms

Information regarding the toxicity of the investigated fungicides against organisms living in aqueous environment as predicted using computational tools is revealed in Table 3. All the computational tools considered in this study indicate that, among the fungicides studied, tetraconazole shows the highest toxicity to fathead minnow and Daphnia magna, followed by metconazole. The ADMETLab2.0 result also indicates that tetraconazole reveals the highest toxicity to Tetrahymena pyriformis, followed again by metconazole. These findings are in good correlation with the results of the experimental study revealing that tetraconazole and metconazole are highly toxic against Lemna minor, but also with other ecotoxicity data indicating at least moderate toxicity of these fungicides against aquatic organisms [59,60].

Flutriafol is the only fungicide investigated that is not considered to produce toxicity against fathead minnow using the admetSAR2.0 computational tool, but this prediction is not sustained by the results obtained using the other computational tools.

Both the experimental results and the predictions obtained in this study show that the studied triazole fungicides produce at least moderate toxicity on several model organisms living in the aqueous environment. Moreover, the specialized literature revealed that high doses of triazole fungicides also affect soil organisms (they decrease the population of earthworms and microbial organisms, disrupting the structure of microbial communities) and usually inhibit the activity of enzymes found in the soil [6,9]. All these data reveal the need for adequate crop management respecting the doses and application intervals of these fungicides (and of all pesticides), so as to avoid or minimize the effects of these fungicides on the soil and the aquatic environment.

### 3.3. Evaluation of the Interactions of Investigated Fungicides with Enzymes Involved in the Photosynthesis Systems, Redox Control and Cellular Detoxification of Lemna minor

**Figure 6:**
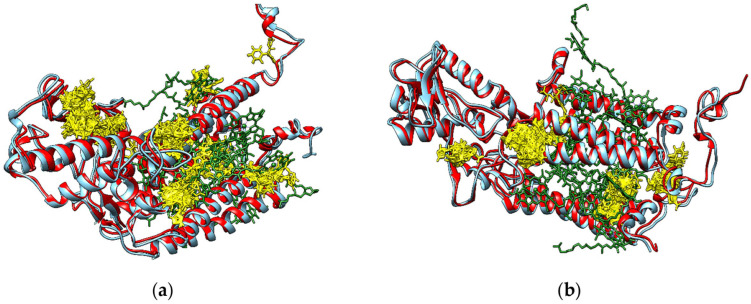
Binding modes of tetraconazole (yellow sticks) to photosystem II C43 (**a**) and C47 (**b**) reaction center proteins (red ribbon) of *Lemna minor*. The blue ribbons illustrate chains C and B, respectively, of the photosystem II CP43 (**a**) and CP47 (**b**) reaction center proteins from *Pisum sativum* bound in the photosystem II complex that are used for identifying the binding sites of chlorophyll a molecules (green sticks).

The interaction energies for the binding of fungicides to enzymes involved in photosynthesis systems, redox control and cellular detoxification of *Lemna minor* resulting from the molecular docking study are shown in Table 4. The binding free energy values reported correspond to the binding modes of the investigated fungicides that best fit the position of the ligand that is present in the crystallographic structure of the protein complex. In the case of enzymes that bind chlorophyll a, fungicides are able to bind to these enzymes in several sites corresponding to the binding of the chlorophyll a molecules (see Figure 6 for tetraconazole), but Table 4 contains, for each fungicide, only the highest value of the interaction energy corresponding to one of these binding sites.

The data presented in Table 4 illustrate that none of the investigated fungicides are able to bind to the active sites of the ribulose bisphosphate carboxylase large chain, of the glutathione peroxidase, and of the ATP synthase subunit alpha, respectively (Supplementary Materials, Figures S2–S4). The highest interacting energies are usually obtained for the interactions of investigated fungicides with photosystem II C43 and C47 reaction center proteins.

Figure 6 reveals that tetraconazole (yellow sticks) is able to bind to photosystem II C43 and C47 reaction center proteins (red ribbon) in numerous sites corresponding to the binding of chlorophyll a molecules (green sticks). The blue ribbon shows the photosystem II reaction center proteins CP43 (a) and CP47 (b) from *Pisum sativum* bound in the photosystem II complex, structures that are used to identify the binding sites of chlorophyll a and beta-carotene molecules. Similar situations are obtained for binding of the other fungicides to the reaction center proteins C43 and C47 of photosystem II (Supplementary Materials, Figures S5 and S6, respectively). The fungicides investigated in this study are also able to bind to the chloroplast ATP synthase subunit beta (Supplementary Materials, Figure S7) in the ATP binding site, to photosystem I P700 chlorophyll a apoprotein A1 (Supplementary Materials, Figure S8) and to photosystem II protein D1 (Supplementary Materials, Figure S9); the binding modes correspond to the sites of chlorophyll a binding, but the interacting energies are smaller than those corresponding to the interactions with photosystem II C43 and C47 reaction center proteins. Bearing in mind the high structural similarities between the photosystem I P700 chlorophyll a apoproteins A1 and A2, and, respectively, between photosystem II proteins D1 and D2, we consider that fungicides are also able to bind to I P700 chlorophyll a apoprotein A2, and, respectively, to photosystem II protein D2. These results are in agreement with published data revealing that pesticides affect photosynthesis by binding to specific sites in the photosystem II complex in plant chloroplasts [34].

**Figure 7:**
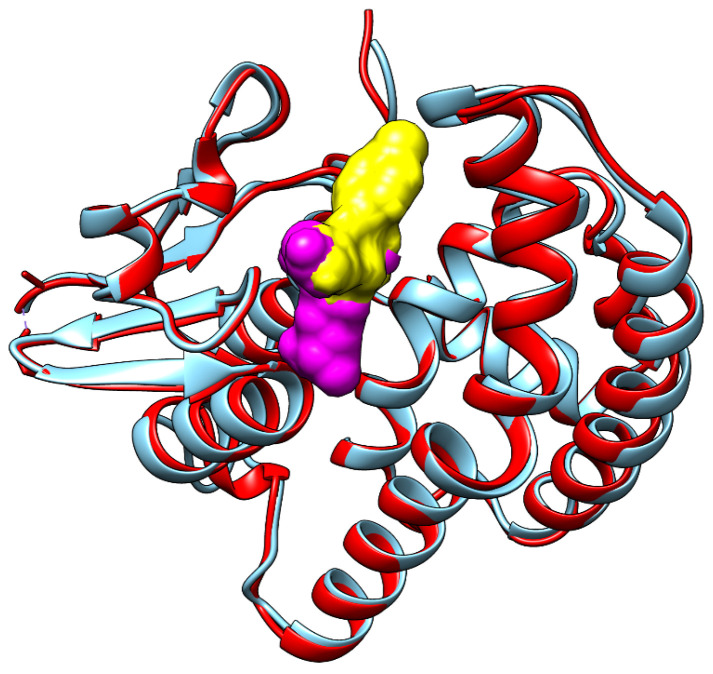
The binding mode with the highest interaction energy of triticonazole (yellow surface) to glutathione S-transferase of *Lemna minor* (red ribbon), corresponding to the binding site of S-hydroxy-glutathione (purple surface) to glutathione transferase from *Alopecurus myosuroides* (blue ribbon).

Another protein that can be inhibited by the investigated fungicides is glutathione S-transferase. The binding energies for this protein are comparable to those corresponding to the binding of fungicides to the C43 and C47 reaction center proteins of photosystem II. Figure 7 shows the binding mode corresponding to the highest interaction energy of triticonazole (yellow surface) to glutathione S-transferase from *Lemna minor* (red ribbon). To identify the binding site for S-hydroxy-glutathione (magenta surface), the structural model for glutathione S-transferase from *Lemna minor* was superposed with the structure of glutathione transferase from *Alopecurus myosuroides* (blue ribbon) in complex with S-hydroxy-glutathione (magenta surface). Triticonazole occupies a region of the binding site of S-hydroxy-glutathione. This result also correlates well with other published data revealing that clotrimazole, another azole fungicide, caused a decrease in glutathione S-transferase activity [20].

There are also several limitations to this study. They are common to computational studies and are reflected by the fact that predictions are based only on the molecular descriptors and models used by the chosen computational tools and do not allow consideration of fungicide concentrations in the aqueous medium. Correlation between the data predicted using distinct models and with other data resulted from experimental studies increases the confidence of the predictions. Also, in this study, the enantioselective effects of the stereoisomers of these fungicides on the enzymes belonging to *Lemna minor* and involved in photosynthesis and cellular detoxification were not addressed. Published data reveal distinct interactions of stereoisomers of triazole fungicides with enzymes found in soil [10,60,64] and with proteins involved in human physiology [10,13]. Accordingly, in our opinion, this issue should be considered and will be the subject of another study.

## 4. Conclusions

In this study, the ecotoxicity of several triazole fungicides against *Lemna minor*, a freshwater plant, was evaluated by determining the EC_50_ for growth inhibition of this plant in the presence of the considered fungicides. All fungicides are toxic according to their classification in aquatic ecotoxicity categories, metconazole and tetraconazole being very toxic; flutriafol, myclobutanil and tebuconazole being moderately toxic; and triticonazole being slightly toxic to *Lemna minor*. Estimated data using the TEST, ADMETLab2.0 and admetSAR2.0 calculation tools show that metconazole and tetraconazole also exhibit high toxicity against fathead minnow, *Daphnia magna* and *Tetrahymena pyriformis*, other aquatic organisms. The results of the molecular docking study suggest that the investigated triazole fungicides affect photosynthesis in *Lemna minor*, because they strongly bind to proteins C43 and C47 in the reaction center of photosystem II, inhibiting the binding of chlorophyll a to these proteins. Another *Lemna minor* enzyme that can be inhibited by these fungicides is glutathione S-transferase, the enzyme involved in cellular detoxification. This study argues that indirect effects of fungicides should be investigated and receive similar attention as direct effects.

## Figures and Tables

**Table 1:** Commercial name and tested concentrations of the six triazole fungicides considered in this study.

Fungicide	Commercial Name	Producer and Country	Tested Concentrations
Flutriafol	Impact	Cheminova A/S, Harboøre, Denmark	0.02, 0.2, 2, 20 and 200 mg/L
Metconazole	Caramba	BASF Agro BV Arnhem, Zürich, Switzerland	0.002, 0.02, 0.2, 2 and 20 mg/L
Myclobutanil	Systhane forte	DowAgroSciences LLC, Indianapolis, IL, USA	0.03, 0.3, 3, 30 and 300 mg/L
Tebuconazole	Sextan	Ascenza Agro S.A., Setúbal, Portugal	0.003, 0.03, 0.3, 3 and 30 mg/L
Tetraconazole	Domark	ISAGRO S.p.A, Milan, Italy	0.005, 0.5, 0.5, 5 and 50 mg/L
Triticonazole	Premis	BASF Agro BV Arnhem, Zürich, Switzerland	0.1, 1, 10, 100 and 1000 mg/L

**Table 2:** AlphaFold models of enzymes belonging to *Lemna minor*, the corresponding structural files of similar enzymes from other plants that are deposited in the Protein Data Bank, and root mean square deviation (RMSD) values for superimposing the AlphaFold models and the crystallographic structures. CA—carbon alpha atoms.

Enzyme Belonging to *Lemna minor*, Its Uniprot and AlphaFold IDs	Corresponding Enzyme Having a Determined Structure in Protein Data Bank, Its PDB and Uniprot IDs	RMSD Values for the Superposition of the AlphaFold Model and Experimental Structure
Enzymes Involved in Photosynthesis		
Chloroplast ATP synthase subunit alpha (A9L981/AF-A9L981-F1)	Chloroplast ATP synthase subunit alpha from *Spinacia oleracea* in complex with ATP (6VMD chain C/P06450)	1.305 Å for 388 CA pruned atom pairs from all 436 atom pairs
Chloroplast ATP synthase subunit beta (A9L9A3/AF-A9L9A3-F1)	Chloroplast ATP synthase subunit beta from *Spinacia oleracea* in complex with ATP (6VMD chain D/P00825)	1.043 Å for 403 CA pruned atom pairs from all 479 atom pairs
Photosystem I P700 chlorophyll a apoproteins A1 (A9L996/AF-A9L996-F1) and A2 (A9L995/AF-A9L995-F1)	Photosystem I P700 chlorophyll a apoprotein A1 from *Pisum sativum* bound in the photosystem I complex and containing beta-carotene, chlorophyll a, and phylloquinone molecules (2WSE chain A/P05310)	0.898 Å for 609 CA pruned atom pairs from all 730 atom pairs
Photosystem II CP43 reaction center protein (A9L992/AF-A9L992-F1)	Photosystem II CP43 reaction center protein from *Pisum sativum* bound in the photosystem II complex and containing chlorophyll a and beta-carotene molecules (6YP7 chain C/P06004)	1.031 Å for 440 CA pruned atom pairs from all 450 atom pairs
Photosystem II CP47 reaction center protein (A9L9C2/AF-A9L9C2-F1)	Photosystem II CP47 reaction center protein from *Pisum sativum* bound in the photosystem II complex containing chlorophyll a and beta-carotene molecules (6YP7 chain B/Q9XQR6)	1.113 Å for 485 CA pruned atom pairs from all 503 atom pairs
Photosystem II proteins D1 (A9L976/AF-A9L976-F1) and D2 (A9L991/AF-A9L991-F1)	Photosystem II protein D1 from *Pisum sativum* bound in the photosystem II complex containing chlorophyll a and beta-carotene molecules (6YP7 chain A/P06585)	1.070 Å for 320 CA pruned atom pairs from all 334 atom pairs
Ribulose bisphosphate carboxylase large chain (A9L9A4/AF-A9L9A4-F1)	Ribulose bisphosphate carboxylase large chain from *Spinacia oleracea* in complex with ribulose-1,5-diphosphate (1RCX chain B/P00875)	0.306 Å for 464 CA pruned atom pairs from all 467 atom pairs
Enzyme Involved in Redox Control		
Glutathione peroxidase (A5Z284/AF-A5Z284-F1)	Glutathione peroxidase from *Schistosoma mansoni* in complex with pyrophosphate (2WGR chain A/Q00277)	0.796 Å for 92 CA pruned atom pairs from all 95 atom pairs
Enzyme Involved in Cellular Detoxification		
Glutathione transferase (A0A0F6PRM5/AF-A0A0F6PRM5-F1)	Glutathione transferase from *Alopecurus myosuroides* in complex with S-hydroxy-glutathione and succinic acid (6RIV chain A/Q9ZS17)	0.690 Å for 202 pruned atom pairs from all 213 atom pairs

**Table 3:** Predicted toxicity against aquatic organisms for the investigated fungicides. NA—non-available data, TEST—Toxicity Estimation Software Tool, FLU—flutriafol, MET—metconazole, MYC—myclobutanil, TEB—tebuconazole, TET—tetraconazole, TRI—triticonazole.

Organism/Fungicide	FLU	MET	MYC	TEB	TET	TRI
TEST						
Fathead minnow LC_50_ 96 h −log_10_ (mg/L)	4.82	4.94	4.78	4.88	5.69	5.57
*Daphnia magna* LC_50_ 48 h −log_10_ (mg/L)	4.44	4.51	5.10	4.50	4.63	4.61
ADMETLab2.0						
Fathead minnow LC_50_ 96 h −log_10_ [(mg/L)/(1000 × MW)]	3.70	4.35	4.15	3.71	5.30	3.73
*Daphnia magna* LC_50_ 48 h −log_10_ [(mg/L)/(1000 × MW)]	4.44	3.89	3.51	3.42	4.64	3.49
*Tetrahymena pyriformis* IGC_50_ 48 h −log_10_ [ (mg/L)/(1000 × MW)]	2.56	3.79	3.11	3.41	4.34	2.96
admetSAR2.0						
Probability to produce 96 h toxicity against fathead minnow	−0.44	0.93	0.97	0.69	0.85	0.98
Probability to produce crustacea 48 h aquatic toxicity	0.61	−0.50	0.69	0.66	0.61	0.55
*Tetrahymena pyriformis* 48 h −log_10_ IGC_50_ (µg/L)	0.40	0.91	1.65	1.38	0.59	1.02

**Table 4:** The highest interacting energies for the binding of investigated fungicides on the enzymes involved in the photosynthesis systems, redox control and cellular detoxification of *Lemna minor*.

Fungicide/Enzyme	Ligand	ΔG (kcal/mol)					
		FLU	MET	MYC	TEB	TET	TRI
Chloroplast ATP synthase subunit alpha	ATP	-	-	-	-	-	-
Chloroplast ATP synthase subunit beta	ADP	−6.25	−6.21	−6.10	−6.77	−6.49	−6.81
Photosystem I P700 chlorophyll a apoproteins A1 and A2	chlorophyll	−7.24	−6.68	−6.42	−6.50	−7.04	−7.08
	phylloquinone a	-	-	-	-	-	-
Photosystem II proteins D1 and D2	chlorophyll a	−6.66	−7.17	−6.17	−7.03	−6.99	−7.23
	β-carotene	-	-	-	-	-	-
	pheophytin a	-	-	-	-	-	-
Photosystem II C43 reaction center protein	chlorophyll a	−7.91	−7.61	−7.99	−7.48	−7.44	−7.66
	β-carotene	-	-	-	-	-	-
Photosystem II C47 reaction center protein	chlorophyll a	−7.65	−7.47	−7.62	−7.44	−8.28	−7.60
	β-carotene	-	-	-	-	-	-
Ribulose bisphosphate carboxylase large chain	ribulose-1,5-diphosphate	-	-	-	-	-	-
Glutathione peroxidase	pyrophosphate 2	-	-	-	-	-	-
Glutathione S-transferase	S-hydroxy-glutathione	−7.38	−7.40	−7.38	−7.99	−7.60	−8.00

## Data Availability

The original contributions presented in the study are included in the article, further inquiries can be directed to the corresponding author/s.

## Supplementary Materials

The following supporting information can be downloaded at: https://www.mdpi.com/article/10.3390/metabo14040197/s1, Figure S1: Superposition of: (a) structures of photosystem I P700 chlorophyll a apoproteins A1 (orange ribbon) and A2 (blue ribbon); (b) photosystem II reaction center proteins D1 (orange ribbon) and D2 (Blue ribbon); Figure S2: Binding modes of flutriafol (a), metconazole (b), myclobutanil (c), tebuconazole (d), tetraconazole (e) and triticonazole (f) to ribulose bisphosphate carboxylase large chain of *Lemna minor* (red ribbon); Figure S3: Binding modes of flutriafol (a), metconazole (b), myclobutanil (c), tebuconazole (d), tetraconazole (e) and triticonazole (f) to gluthatione peroxidase of *Lemna minor* (red ribbon); Figure S4: Binding modes of flutriafol (a), metconazole (b), myclobutanil (c), tebuconazole (d), tetraconazole (e) and triticonazole (f) to chloroplast ATP synthase subunit alpha of *Lemna minor* (red ribbon); Figure S5: Binding modes of flutriafol (a), metconazole (b), myclobutanil (c), tebuconazole (d), and triticonazole (e) to photosystem II CP43 reaction center protein of *Lemna minor* (red ribbon); Figure S6: Binding modes of flutriafol (a), metconazole (b), myclobutanil (c), tebuconazole (d), and triticonazole (e) to photosystem II CP47 reaction center protein of *Lemna minor* (red ribbon); Figure S7: Binding modes of flutriafol (a), metconazole (b), myclobutanil (c), tebuconazole (d), tetraconazole (e) and triticonazole (f) to chloroplast ATP synthase subunit beta of *Lemna minor* (red ribbon); Figure S8: Binding modes of flutriafol (a), metconazole (b), myclobutanil (c), tebuconazole (d), tetraconazole (e) and triticonazole (f) to photosystem I P700 chlorophyll a apoprotein A1 of *Lemna minor* (red ribbon); Figure S9: Binding modes of flutriafol (a), metconazole (b), myclobutanil (c), tebuconazole (d), tetraconazole (e) and triticonazole (f) to photosystem II protein D1 of *Lemna minor* (red ribbon).
