# Supplementary material for: Evaluation of the Aquatic Toxicity of Several Triazole Fungicides
Source: Metabolites. 2024 Apr 2;14(4):197. doi: 10.3390/metabo14040197 (PMC11051906; doi:10.3390/metabo14040197)
Supplement: Supplementary file 1 [file metabolites-14-00197-s001.zip › metabolites-2897533-supplementary.pdf]

metabolites

MDPI

Supplementary material

Evaluation of the Aquatic Toxicity of Several Triazole Fungicides

Bianca-Vanesa Boros [1,2,†], Diana-Larisa Roman [1,2,†] and Adriana Isvoran [1,2,*,†]

1 Department of Biology-Chemistry, Faculty of Chemistry, Biology, Geography, West University of Timisoara, 16 Pestalozzi, 300115 Timisoara, Romania; bianca.boros@e-uvt.ro (B.-V.B.); diana.roman@e-uvt.ro (D.-L.R.)
2 Advanced Environmental Research Laboratories (AERL), 4 Oituz, 300086 Timisoara, Romania
* Correspondence: adriana.isvoran@e-uvt.ro
† These authors contributed equally to this work.

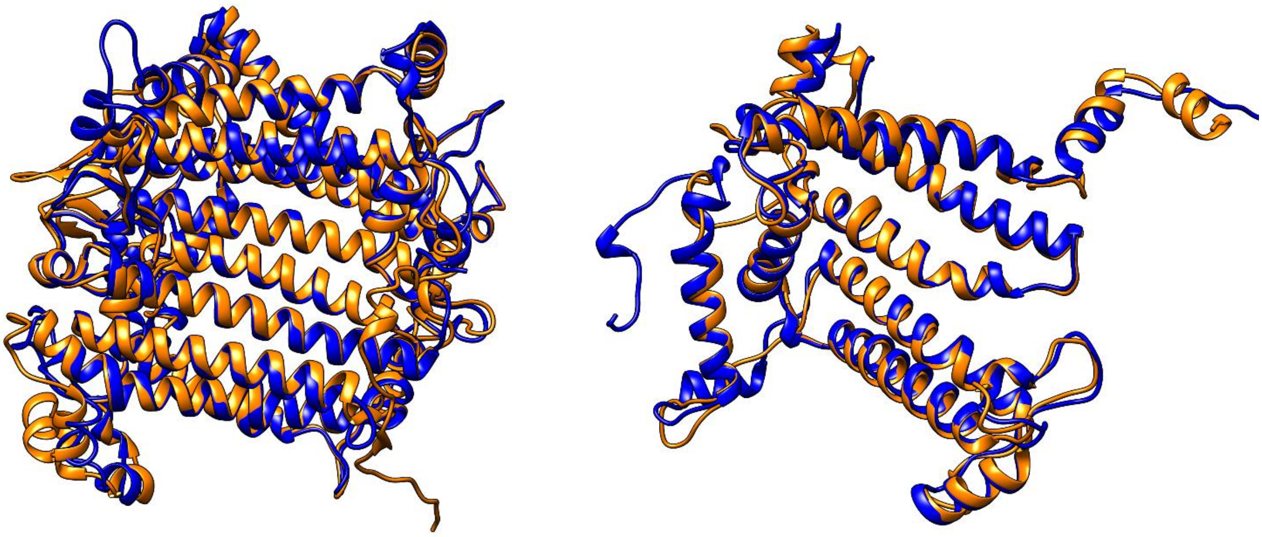

(a) (b)

Figure S1. Superposition of: (a) structures of photosystem I P700 chlorophyll a apoproteins A1 (orange ribbon) and A2 (blue ribbon); (b) photosystem II reaction center proteins D1 (orange ribbon) and D2 (Blue ribbon).

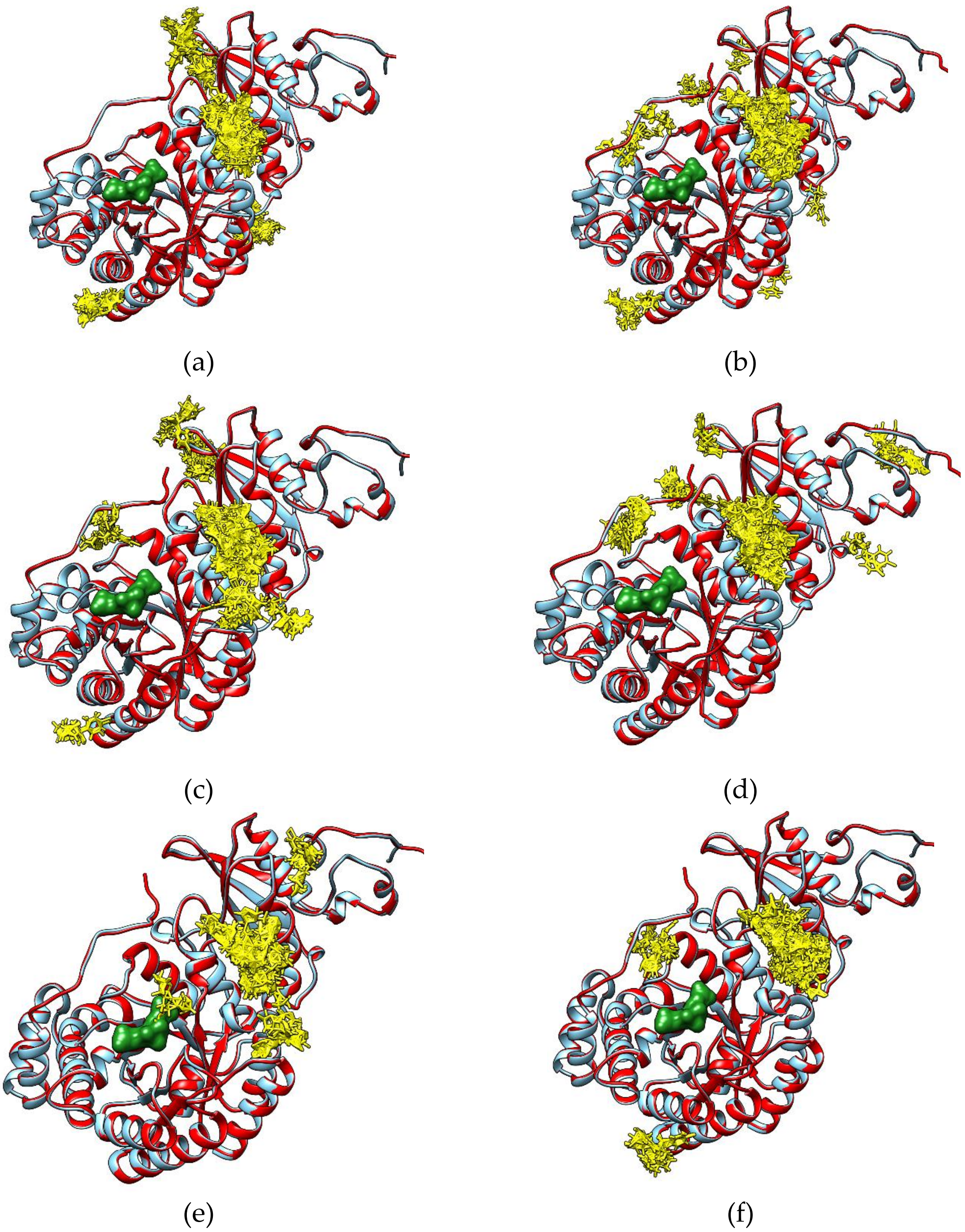

(a) (b) (c) (d) (e) (f)

Figure S2. Binding modes of flutriafol (a), metconazole (b), myclobutanil (c), tebuconazole (d), tetraconazole (e) and triticonazole (f) to ribulose bisphosphate carboxylase large chain of *Lemna minor* (red ribbon). To identfy the active site of the enzyme, it was superposed to the structure of ribulose bisphosphate carboxylase large chain from *Spinacia oleracea* (blue ribbon) in complex with ribulose-1,5-diphosphate (green surface). Binding modes of fungicides are revealed as yellow sticks.

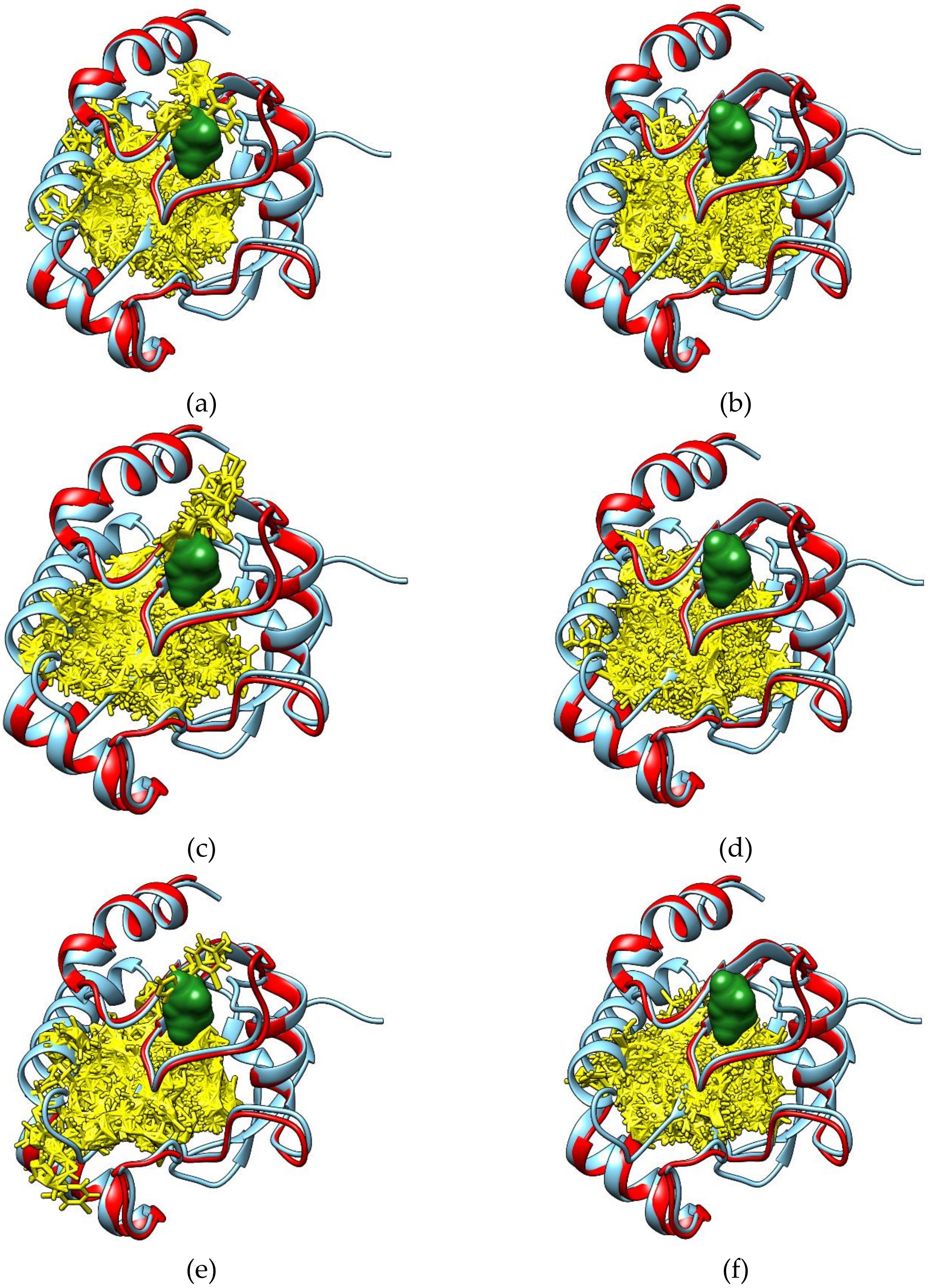

Figure S3. Binding modes of flutriafol (a), metconazole (b), myclobutanil (c), tebuconazole (d), tetraconazole (e) and triticonazole (f) to gluthathione peroxidase of *Lemna minor* (red ribbon). To identfy the active site of the enzyme, it was superposed to the structure of glutathione peroxidase from *Schistosoma mansoni* (blue ribbon) in complex with pyrophosphate (green surface). Binding modes of fungicides are revealed as yellow sticks.

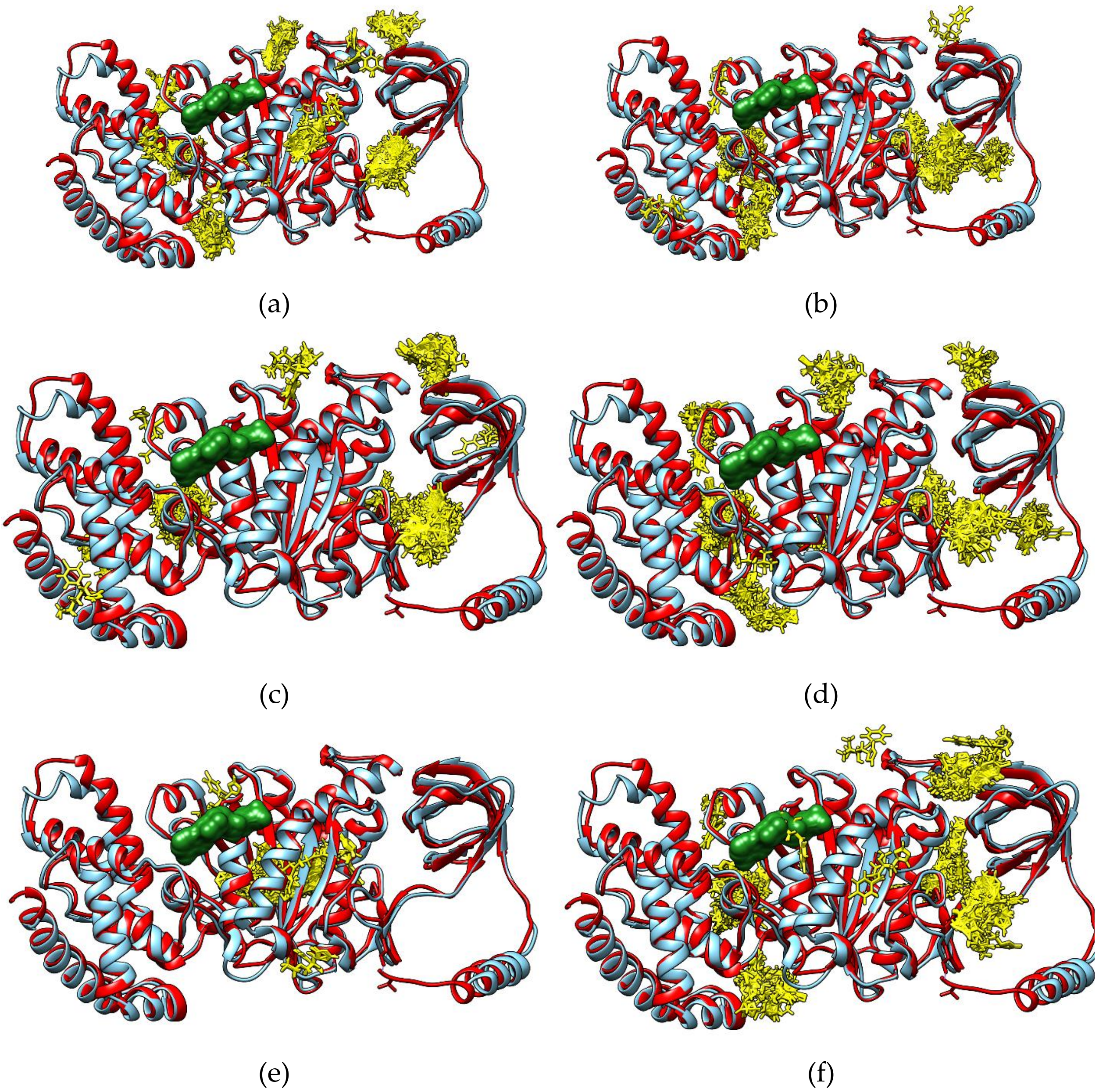

Figure S4. Binding modes of flutriafol (a), metconazole (b), myclobutanil (c), tebuconazole (d), tetraconazole (e) and triticonazole (f) to chloroplast ATP synthase subunit alpha of *Lemna minor* (red ribbon). To identfy the active site of the enzyme, it was superposed to the structure of chloroplast ATP synthase subunit alpha from *Spinacia oleracea* (blue ribbon) in complex with ATP (green surface). Binding modes of fungicides are revealed as yellow sticks.

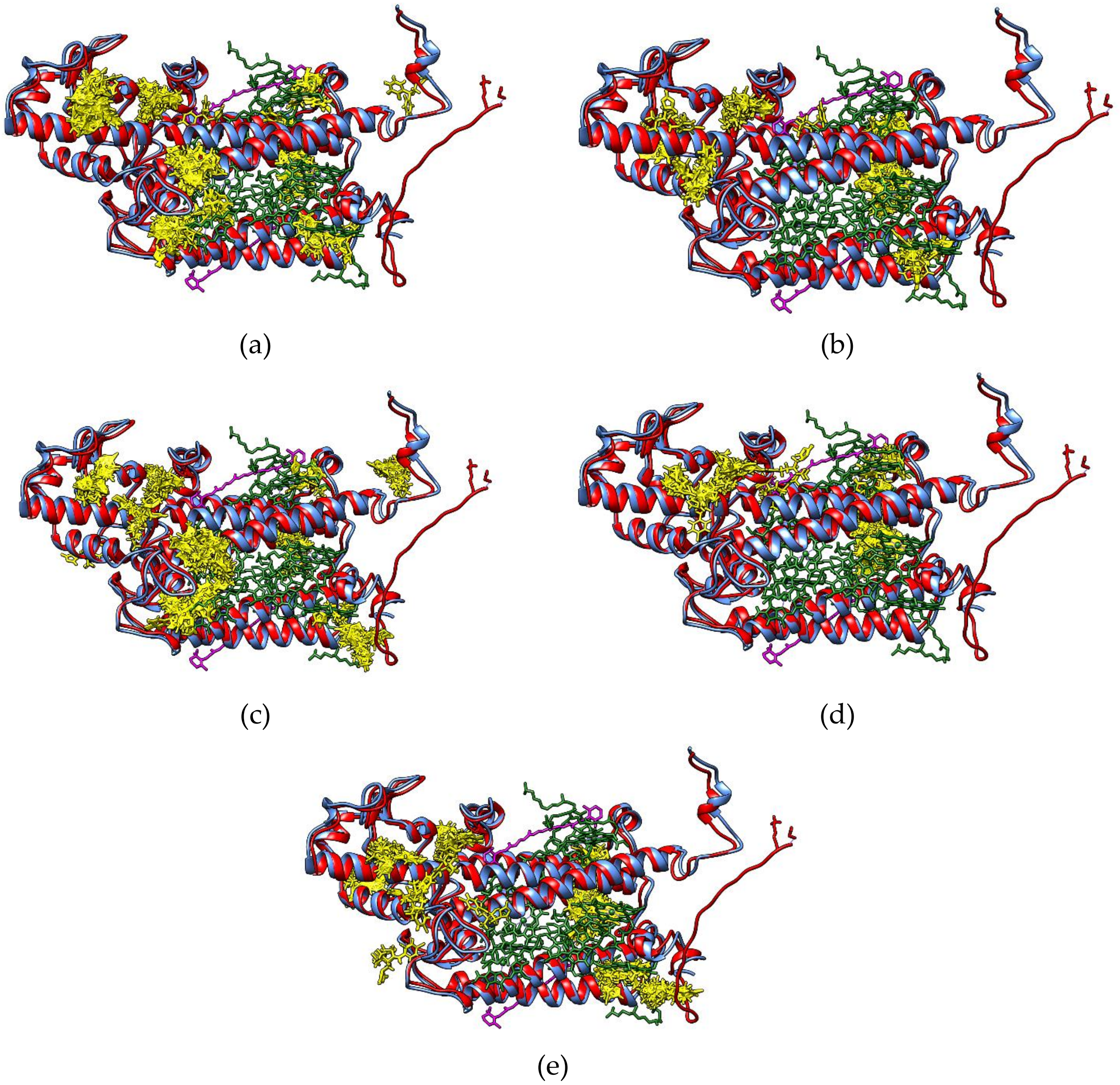

(a) (b) (c) (d) (e)

Figure S5. Binding modes of flutriafol (a), metconazole (b), myclobutanil (c), tebuconazole (d), and triticonazole (e) to photosystem II CP43 reaction center protein of *Lemna minor* (red ribbon). To identfy the active site of the enzyme, it was superposed to the structure of photosystem II CP43 reaction center protein from *Pisum sativum* (blue ribbon) bound in the photosystem II and containing chlorophill a (green sticks) and beta-carotene (magenta sticks) molecules. Binding modes of fungicides are revealed as yellow sticks.

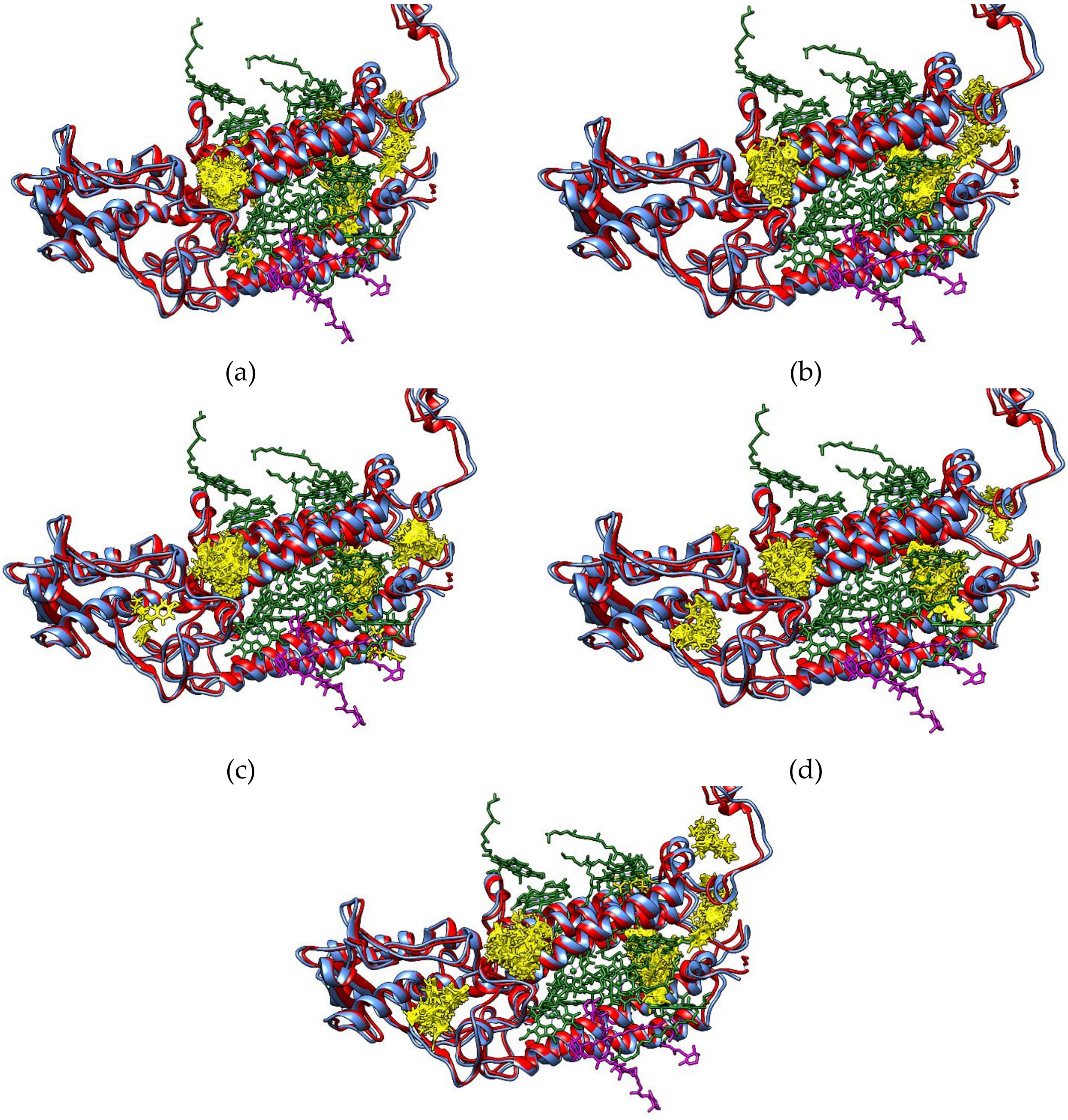

(e)

Figure S6. Binding modes of flutriafol (a), metconazole (b), myclobutanil (c), tebuconazole (d), and triticonazole (e) to photosystem II CP47 reaction center protein of *Lemna minor* (red ribbon). To identfy the active site of the enzyme, it was superposed to the structure of photosystem II CP47 reaction center protein from *Pisum sativum* (blue ribbon) bound in the photosystem II and containing chlorophyll a (green sticks) and beta-carotene (magenta sticks) molecules. Binding modes of fungicides are revealed as yellow sticks.

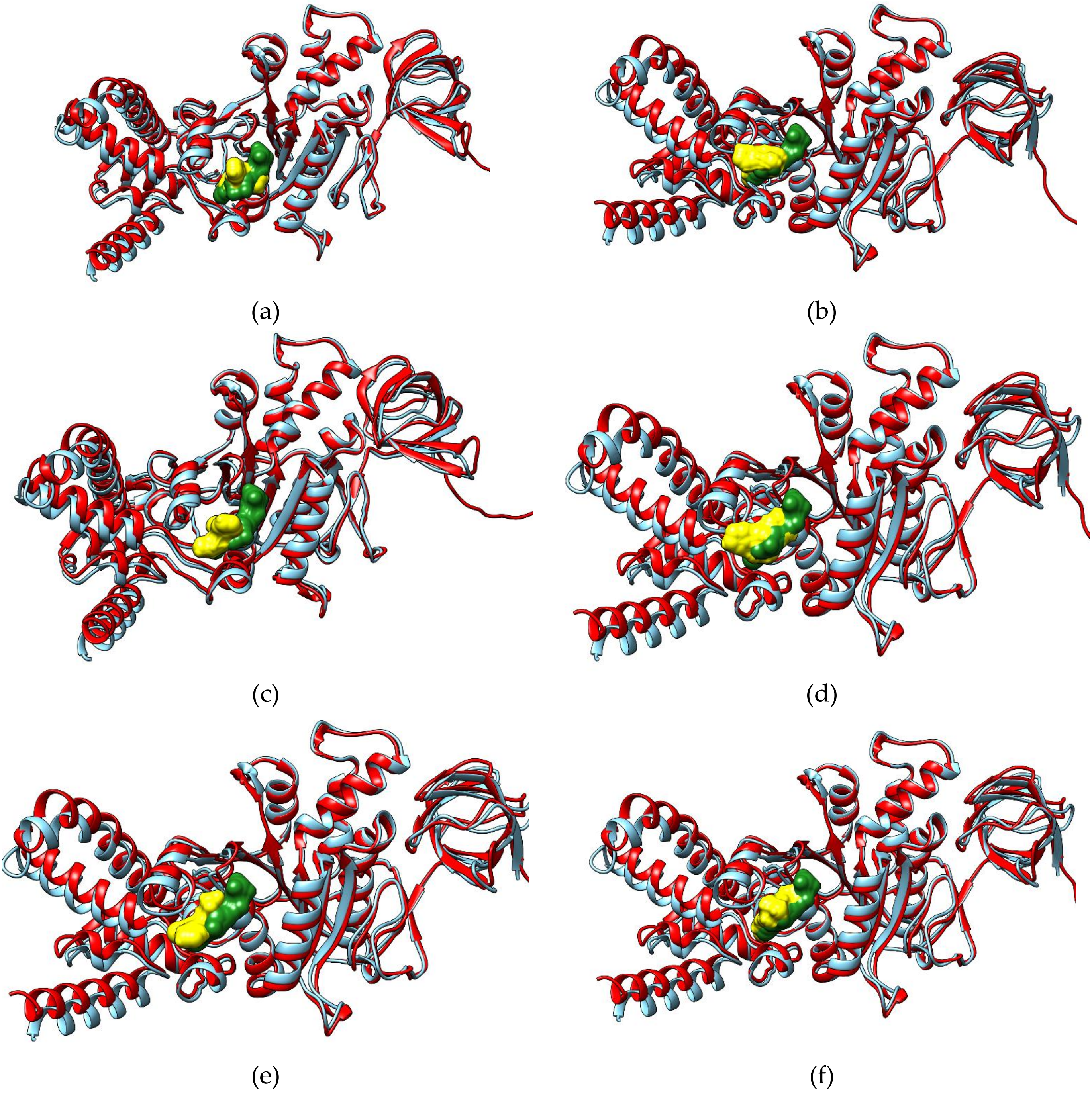

(a) (b) (c) (d) (e) (f)

Figure S7. Binding modes of flutriafol (a), metconazole (b), myclobutanil (c), tebuconazole (d), tetraconazole (e) and triticonazole (f) to chloroplast ATP synthase subunit beta of *Lemna minor* (red ribbon). To identfy the active site of the enzyme, it was superposed to the structure of chloroplast ATP synthase subunit beta from *Spinacia oleracea* (blue ribbon) in complex with ADP (green surface). Binding modes of fungicides are revealed as yellow surface.

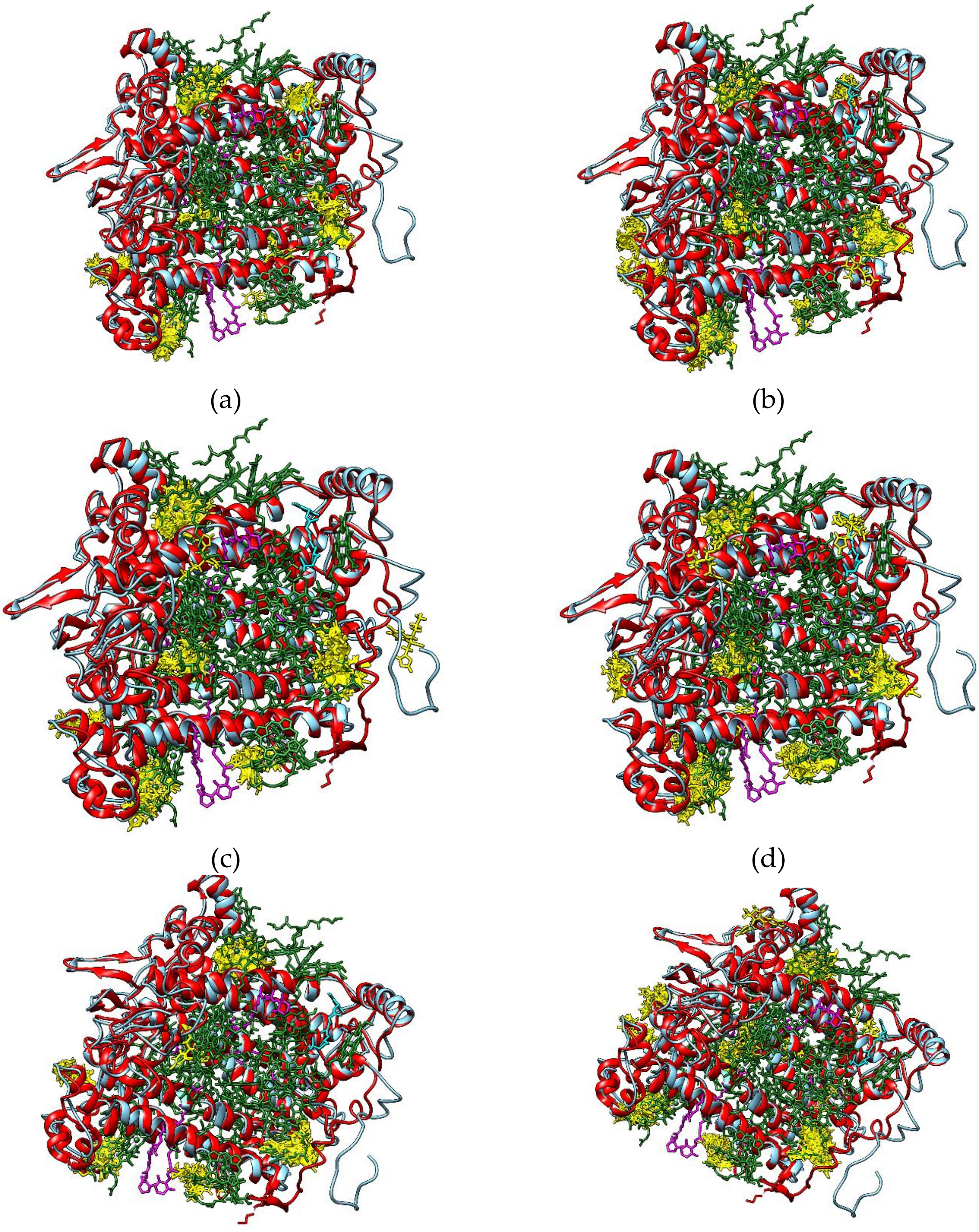

Figure S8. Binding modes of flutriafol (a), metconazole (b), myclobutanil (c), tebuconazole (d), tetraconazole (e) and triticonazole (f) to photosystem I P700 chlorophyll a apoprotein A1 of *Lemna minor* (red ribbon). To identfy the active site of the enzyme, it was superposed to the structure of photosystem I P700 chlorophyll a apoprotein A1 from *Pisum sativum* (blue ribbon) bound in the photosystem I complex and containing chlorophyll a (green sticks), beta-carotene (magenta sticks) and phylloquinone (cyan sticks) molecules. Binding modes of fungicides are revealed as yellow sticks.

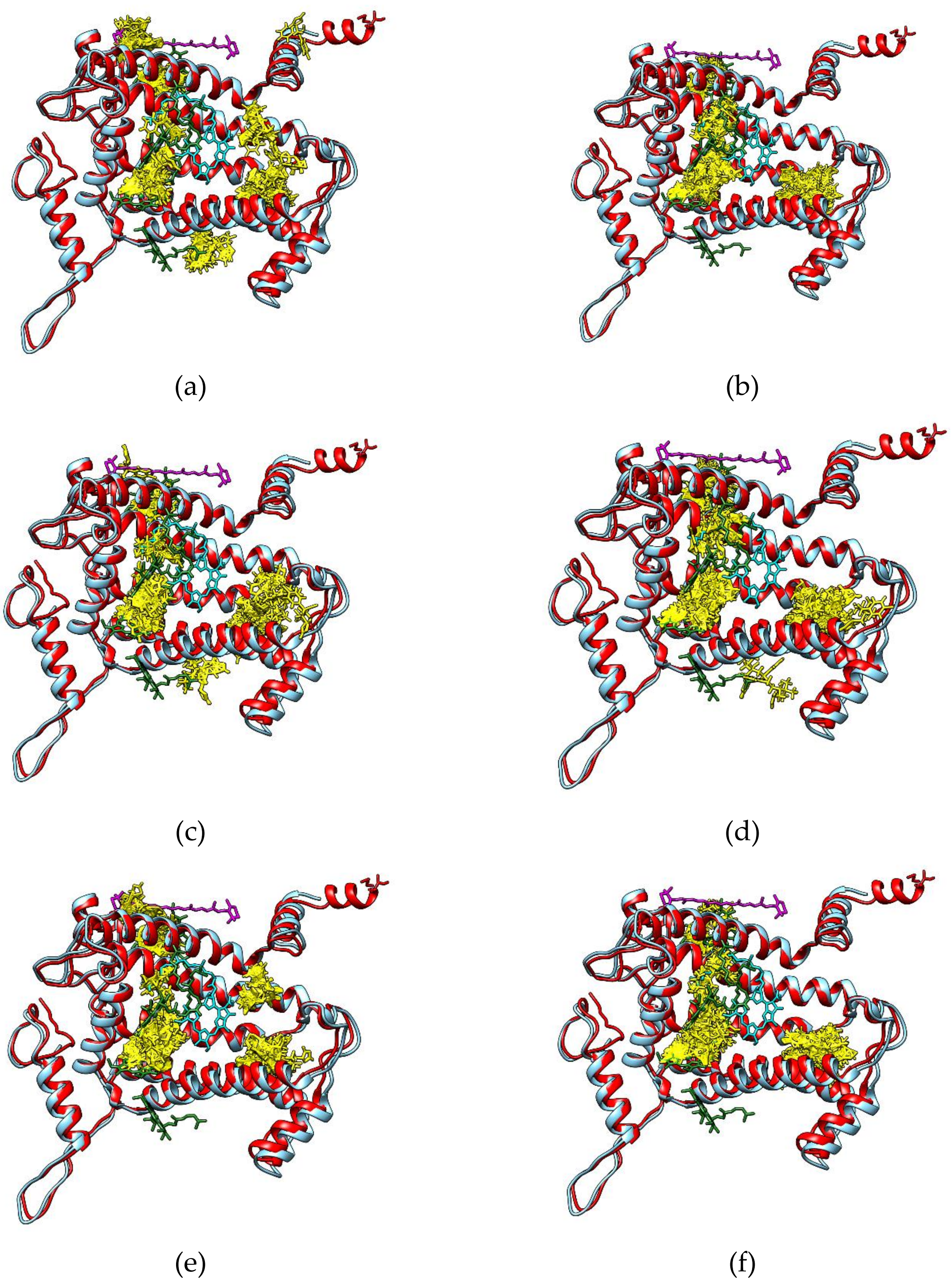

Figure S9. Binding modes of flutriafol (a), metconazole (b), myclobutanil (c), tebuconazole (d), tetraconazole (e) and triticonazole (f) to photosystem II protein D1 of *Lemna minor* (red ribbon). To identfy the active site of the enzyme, it was superposed to the structure of photosystem II protein D1 from *Pisum sativum* (blue ribbon) bound in the photosystem I complex and containing chlorophyll a (green sticks), beta-carotene (magenta sticks), and pheophytin a (cyan sticks). Binding modes of fungicides are revealed as yellow sticks.
